# Effects of *TGF-β1* and *VEGF-A* transgenes on the osteogenic potential of bone marrow stromal cells in vitro and in vivo

**DOI:** 10.1177/2041731412459745

**Published:** 2012-09-04

**Authors:** Shinji Kuroda, Dale R Sumner, Amarjit S Virdi

**Affiliations:** 1 Department of Masticatory Function Rehabilitation, Tokyo Medical and Dental University, Japan; 2 Department of Anatomy and Cell Biology, Rush University Medical Center, USA

**Keywords:** Gene transfer, bone marrow, TGF-β1, VEGF-A, tissue engineering

## Abstract

An exogenous supply of growth factors and bioreplaceable scaffolds may help bone regeneration. The aim of this study was to examine the effects of *TGF-β1* and *VEGF-A* transgenes on the osteogenic potential of bone marrow stromal cells. Rat bone marrow stromal cells were transfected with plasmids encoding mouse TGF-β1 and/or VEGF-A complementary DNAs and cultured for up to 28 days. Furthermore, collagen scaffolds carrying combinations of the plasmids-transfected cells were implanted subcutaneously in rats. The transgenes increased alkaline phosphatase activity, enhanced mineralized nodule formation, and elevated osteogenic gene expressions in vitro. In vivo, messenger RNA expression of osteogenic genes such as *BMP*s and *Runx2* elevated higher by the transgenes. The data indicate that exogenous TGF-β1 and VEGF-A acted synergistically and could induce osteoblastic differentiation of bone marrow stromal cells in both cell culture and an animal model. The results may provide valuable information to optimize protocols for transgene-and-cell-based tissue engineering.

## Introduction

Osteoblastic differentiation is essential for bone healing; the regenerative process involves the continual generation of osteoblasts and bone formation after the terminal differentiation of these cells. The supply of osteoblasts is sustained by an abundance of mesenchymal stem cells and intermediate osteoprogenitor cells.^1,2^ However, the cell repositories, which can elicit cell proliferation and differentiation important for osteogenesis, are still to be defined. The reservoir of mesenchymal stem cells and osteoprogenitor cells may lie within the bone marrow, periosteum, and muscle connective tissue,^3–7^ for instance, cells derived from these tissues mineralize when cultured in vitro in the presence of ascorbic acid, β-glycerol phosphate, and dexamethasone^8–10^ and contribute to bone formation when transplanted into ectopic sites in vivo. Although the mechanism by which these cells proceed to osteoblastic differentiation remains unclear, they could play roles in normal bone homeostasis.

The use of endogenous stem or progenitor cells within predesigned matrix scaffolds along with cytokines or growth factors for local delivery has presented alternative strategies for more effective tissue engineering.^11,12^ In particular, growth factors promoting spinal fusion have been increasingly investigated for their capacity to enhance bone regeneration. However, several barriers to effective and safe local delivery of such proteins in vivo have been identified^13^: their very short life, high production cost, and inability to maintain full bioactivity after their incorporation into controlled delivery systems. Moreover, high-dose local delivery can be associated with both local and systemic toxicities. Gene therapy is an effective approach by which therapeutic proteins can be delivered in a physiological and persistent manner.^14,15^ One method of gene delivery is to use plasmids to carry cytokine and growth factor genes. Plasmid DNA possesses a stable and flexible chemistry and does not cause systemic toxicity because of the high efficiency of DNA catabolism in the bloodstream.^16^

Transforming growth factor-beta 1 (TGF-β1), the largest source of which is bone,^17^ has been implicated in osteoblast proliferation and differentiation^18,19^ and is expressed at high levels during bone growth and development with an adequate blood supply.^17,20–24^ Vascular endothelial growth factor (VEGF), which is secreted by many cells including osteoblasts and osteoblast-like cells, plays an important role for adequate angiogenesis^25,26^ and may be intimately related to bone development and fracture healing because both intramembranous and endochondral ossifications are associated with capillary development.^27,28^ VEGF expression is regulated by several growth factors, hormones, and cytokines, such as TGF-β1.^29^ These two proteins are associated with osteogenesis during bone growth, development, and healing; but they do not stimulate stem cells or bone progenitor cells to generate to be osteoblasts as directly as bone morphogenetic proteins (BMPs). However, these proteins have efficacy on not only cell migration and propagation but also on angiogenesis indispensable for bone formation. Furthermore, cells stimulated and proliferated by these growth factors could produce and secrete more BMPs to osteoblastic progenitor cells and preosteoblasts for osteoblastic maturation. Although many growth factors including TGF-β1and VEGF have been examined in vitro and in vivo in studies of cellular or molecular events, the combination of TGF-β1 and VEGF, especially in vivo, has rarely been investigated.

In this study, we aimed to examine the effects of *TGF-β1* and *VEGF-A* transgenes on the osteogenic potential of bone marrow stromal cells in culture and in implanted bioreplaceable scaffolds in vivo. We introduced transgenes of mouse TGF-β1 (mTGF-β1), mouse VEGF-A (mVEGF-A), and an mTGF-β1/mVEGF-A combination in rat bone marrow stromal cells by lipofection using plasmid DNA and investigated their effects on cell proliferation, osteoblastic differentiation, and osteogenic gene expressions.

## Materials and methods

The study protocol was approved by the institutional Recombinant DNA Experiment Committee and Animal Experiment Committee. All the experimental procedures were performed in accordance with the institutional guidelines for the care and manipulation of laboratory animals.

### Plasmids encoding mTGF-β1 and mVEGF-A genes

pGT-mTGF-β1 plasmid (7888 bp) and pGT-mVEGF-A plasmid (7362 bp), encoding mTGF-β1 complementary DNA (cDNA) (570 bp; full length) and mVEGF-A cDNA (1170 bp; full length), respectively, were purchased from Invitrogen. Each plasmid included the hEF1-HTLV promoter for transcription of the genes in the transfected cells. The plasmids were adequately cloned and purified by using a Plasmid Purification Kit (QIAGEN).

### Harvest and culture of bone marrow stromal cells

Bone marrow stromal cells were sourced from male Sprague Dawley rats (400 g). After the animals were euthanized in a CO_2_ chamber, their femurs were resected and bone marrow stromal cells were obtained from the distal femurs by flushing the marrow cavities. The cells were seeded in Dulbecco’s modified Eagle’s medium (DMEM) containing 10% fetal bovine serum (FBS) and 1% penicillin–streptomycin in 75-cm^2^ flasks. Then, only the primary culture medium received 1.25 µg/mL amphotericin B (Fungizone; Gibco-BRL). The cells were repeatedly trypsinized and passaged in DMEM containing 10% FBS and 1% penicillin–streptomycin while growing confluent. At the fourth passage, the cells were plated at 1 × 10^5^/well in 24-well plates.

### Plasmid-based gene transfer

The plated cells were divided into four groups according to the type of transgene: the mTGF-β1 and mVEGF-A transgenic groups received the pGT-mTGF-β1 and pGT-mVEGF-A plasmids, respectively; the mTGF-β1/mVEGF-A transgenic group received a combination of the pGT-mTGF-β1 and pGT-mVEGF-A plasmids; and the nontransgenic (control) group received a vehicle vector. Both the plasmids encoded the enhanced green fluorescent protein (EGFP) gene, which is transcribed by the human cytomegalovirus immediate early promoter and first intron A (hCMV-IA) promoter and translated to the protein in the cytoplasm. After 24 h of culture, when the cell number reached semiconfluency, the medium was replaced with fresh one with no antibiotics, followed by lipofection with 1 µg plasmid carrying the m*TGF-β1* or m*VEGF-A* gene, or with 2 µg plasmid of the combination (1 µg each) using Lipofectamine (Invitrogen). The cells trypsinized at days 1, 2, 4, 7, 14, and 28, were suspended in 100 µL phosphate-buffered saline (PBS) containing 0.1% *t*-octylphenoxypolyethoxyethanol (Triton X-100; Sigma) in 1.5 mL eppendorf tubes under ultrasound, and centrifuged. The supernatant was directly processed for EGFP measurement at 488 nm (excitation) and 509 nm (emission).

### Alkaline phosphatase-positive cell staining and mineralized nodule staining

Naphthol AS-MX phosphate (0.1 mg/mL) and fast blue BB salt (0.6 mg/mL) were dissolved in Tris-HCl buffer (0.1 M, pH 8.8) containing *N,N*-dimethylformamide (0.5%) and MgCl_2_ (2 mM) and filtrated to serve as the alkaline phosphatase (ALP)-positive cell-staining solution. The cells trypsinized at days 7, 14, 21, and 28 were washed twice with PBS and fixed in 3.7% formalin for 10 min. After the fixed cells were rinsed with PBS twice, PBS was replaced with 1 mL of the staining solution and the cells were incubated at 37°C for 20 min until the ALP-positive cells stained blue. The staining reaction was stopped by washing with PBS. Digital images were captured with a microscope.

Then, the same cells were used for examining mineralized nodule. The staining solution in this case was prepared by dissolving alizarin red S (1%) in 1/100 water-diluted ammonium hydroxide and filtration. The cells were washed twice with PBS and immersed in methanol for 10 min. After tapping in water, the cells were incubated for 2 min with 500 µL of the staining solution per well until mineralized nodules appeared red. The staining reaction was terminated by washing with water. Digital images were captured with a microscope.

### Fluorometric assay

Hoechst 33258 dye (Polysciences) was used for the assay to estimate the DNA amount. Cultured bone marrow stromal cells from three wells per group were trypsinized, suspended with 100 µL PBS containing 0.1% Triton X-100 (Sigma) in 1.5 mL eppendorf tubes under ultrasound, and centrifuged at days 1, 2, 4, 7, 14, and 28. The cell pellets were dissolved ready for chemical reaction in 2-mL Hoechst dye buffer (10 mM Tris, 100 mM NaCl, 1 mM ethylenediaminetetraacetic acid (EDTA), pH 7.4). Then, 100 µL of each sample was distributed in a well of a 96-well plate, and 100 µL of Hoechst dye (1 µg/mL in Hoechst dye buffer) was added to each well. The fluorescence of the dye/sample complex was immediately measured at an excitation wavelength of 360 nm and an emission wavelength of 460 nm by a Bio-Tek FL500 fluorescence microplate reader (BioTek Instruments, Inc., USA).

### Measurement of ALP activity

ALP yellow liquid substrate (Sigma) containing *p*-nitro-phenylphosphate was used for the assay. The cells were collected in the same manner at days 1, 2, 4, 7, 14, and 28 and directed to chemical reaction. Thereafter, 10 µL of each sample was placed in a well of a 96-well plate and 200 µL of the yellow substrate was added to each well. After incubation at 37°C in a dark chamber for 30 minutes, the reaction was terminated by adding 50 µL of sodium hydroxide (3 N). The yellow reaction product was read at 405 nm.

### Effect of dexamethasone on mineralized nodule formation

Dexamethasone (10^−8^ M), β-glycerol phosphate (10 mM), and ascorbic acid (50 µg/mL) were added to the culture medium to serve as a conditioned medium for osteogenesis 24 h after lipofection. The conditioned medium was replaced with fresh medium every 7 days. At days 7, 14, and 28, the cultured cells were fixed in methanol and rinsed with deionized water. The mineralized nodules were stained with 1% alizarin red S (Sigma).

### Real-time polymerase chain reaction analysis

The expressions of 15 genes related to osteogenesis were analyzed by real-time polymerase chain reaction (PCR). The primer pairs were designed by using Primer3 software (Table 1). Bone marrow cells from three wells per group were pooled and homogenized in TRIzol buffer (Invitrogen) at days 1, 2, 4, 7, and 14, followed by the extraction of total RNA using the SuperScript First-Strand Synthesis System for reverse transcription polymerase chain reaction (RT-PCR) (Invitrogen). The reverse-transcribed cDNAs were amplified by a Taq polymerase system with their compatible primer sets using the SmartCycler (Cepheid). The amounts of the PCR products during the PCR cycles were visualized as fluorescence curves of SYBR Green dye (Molecular Probes), which binds to PCR products in every cycle, and cycle threshold (Ct) was determined as the PCR cycle number at which the fluorescence reached the threshold. The expression of each gene at a time point was calculated relative to its expression in the control group at the same time point, after normalization to GAPDH expression in each group (Table 1). The expressions of the m*TGF-β1* and m*VEGF-A* genes detected by real-time PCR analysis reflected the dilution of the carried plasmids during cell division.

**Table 1.** PCR primer sets and formula to calculate relative amount of gene expression of interest to GAPDH gene expression

Gene	Fw	Rv	Product size	Ta
*GAPDH*	aactcccattcctccacctt	Gagggcctctctcttgctct	200	57.1
*ALP*	gagcaggaacagaagtttgc	Gttgcagggtctggagagta	202	57.1
*Osteopontin*	gatgaaccaagcgtggaaac	Tgaaactcgtggctctgatg	200	57.1
*Osteocalcin*	agctcaaccccaattgtgac	Agctgtgccgtccatacttt	190	57.1
*Collagen I*	ttgaccctaaccaaggatgc	Caccccttctgcgttgtatt	197	57.1
*Collagen II*	gtacactgccctgaaggatg	Attgtgttgttttggggttg	201	53.1
*VEGF*	ttgagaccctggtggacatc	Ctcctatgtgctggctttgg	192	59.2
*bFGF*	gtcccctgtggtagagcttg	Ccagcaggaatgcttgaagt	197	57.1
*Cbfa1*	gccaggttcaacgatctgag	Gaggcggtcagagaacaaac	201	59.2
*IGF-1*	tgtggatgagtgttgcttcc	Gggaggctcctcctacattc	202	57.1
*TGF-β1*	caacaattcctggcgttacc	Tgggactgatcccattgatt	200	55.1
*BMP-2*	aaggcaccctttgtatgtgg	Catgccttagggattttgga	189	55.1
*BMP-4*	agcgtagtcccaagcatcac	Acaatggcatgattggttga	199	53.0
*RANK*	gccagcaagaagtgtgtgaa	Ccggtccgtgtactcatctt	212	59.2
*RANKL*	ccagcatcaaaatcccaagt	Tgaaagccccaaagtacgtc	201	55.1
*Osteoprotegerin*	gaatggtcactgggctgttt	Ctggcagctttgcacaatta	197	55.1

Fw: sequences of forward primer; Rv: sequences of reverse primer; Ta: annealing temperature.

2^-(Ct-Ct_GAPDH_)^; Ct: threshold cycle.

### Implantation of mTGF-β1 and mVEGF-A transgenic bone marrow cells

As in the in vitro experiment, rat bone marrow stromal cells were obtained, cultured, divided into four groups, and transfected with the pGT-mTGF-β1 plasmid, pGT-mVEGF-A plasmid, combination of the pGT-mTGF-β1 and pGT-mVEGF-A plasmids, or a vehicle vector. The prepared cells were cocultured with collagen sponges (BD 3D Collagen Composite Scaffold; BD Biosciences), while being gently rotated to allow even distribution of the cells inside the scaffolds, in 5 mL DMEM with 10% FBS and 1% penicillin–streptomycin overnight. Then, the matrices of the four transgenic groups along with those of a group using a collagen sponge without cells were subcutaneously implanted in the backs of Sprague Dawley rats (4–6 months old). After the specimens developed in the animals for 2 weeks, the neotissues from every four animals were excised and subjected to architectural assessment by microcomputed tomography (µCT), histological examination by toluidine blue staining, and gene expression analysis by RT-PCR, respectively.

## Results

### Detection of transfection

**Figure 1.**
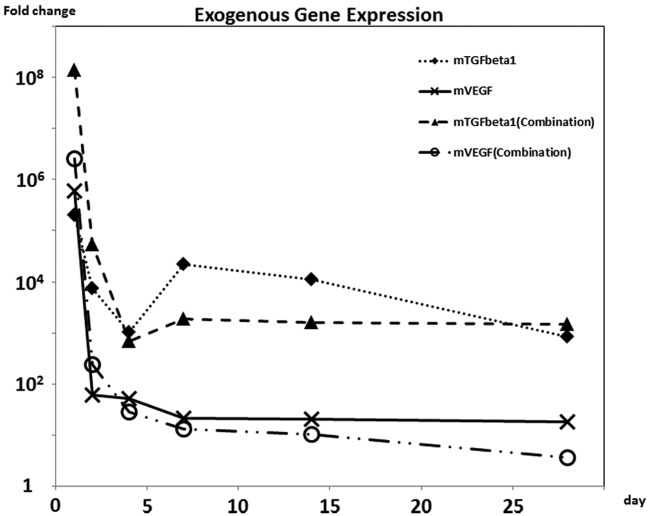
Efficiency of the gene transfer. PCR: polymerase chain reaction. The mouse genes that were transfected into rat bone marrow cells were monitored by fluorescence dye in real-time PCR and expressed on a logarithmic scale as fold changes to those in the control culture.

The intensity of EGFP fluorescence diminished time dependently and faded by day 7 (data not shown). Real-time PCR analysis represented the m*TGF-β1* and m*VEGF-A* gene expressions from the pGT-mTGF-β1 and pGT-mVEGF-A plasmids, respectively, in all the transgenic groups, which attenuated quickly by day 7 (Figure 1).

### Altered cell phenotype in culture

**Figure 2.**
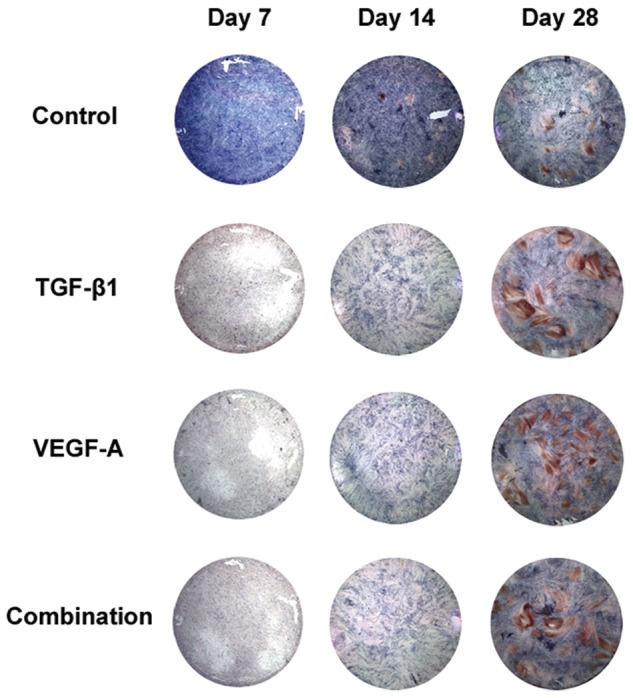
ALP-positive cell staining and mineralized nodule formation. ALP: alkaline phosphatase; PBS: phosphate-buffered saline; TGF-β1: transforming growth factor-beta 1; VEGF-A: vascular endothelial growth factor A. Cultured transgenic cells were washed twice with PBS, fixed in 3.7% formalin for 10 min, and rinsed with PBS twice. ALP-positive cell staining was performed with Naphthol AS-MX phosphatase (0.1 mg/mL) and fast blue BB salt (0.6 mg/mL) at 37°C for 20 min. The staining reaction was stopped by washing with PBS. Subsequently, the cells were immersed in methanol for 10 min and tapped in water. Finally, they were stained with alizarin red S (1%) solution for 2 min. The staining reaction was terminated by washing with water.

**Figure 3.**
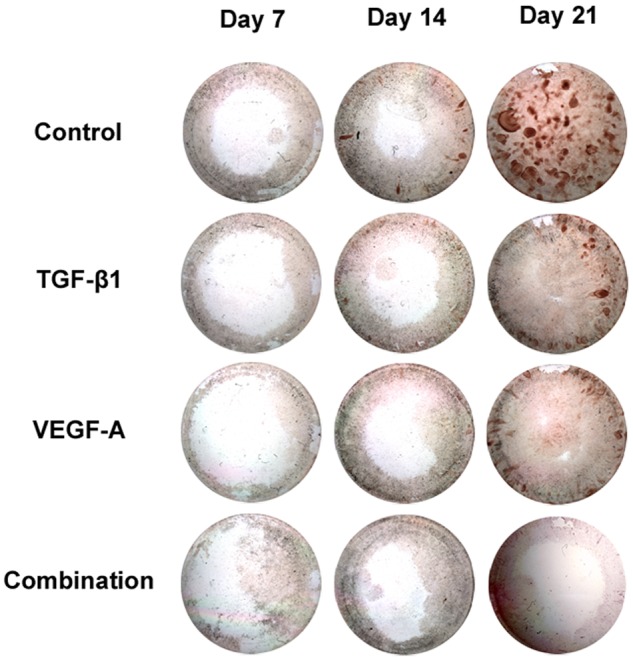
Effects of dexamethasone on the transgenic cells. DMEM: Dulbecco’s modified Eagle’s medium; TGF-β1: transforming growth factor-beta 1; VEGF-A: vascular endothelial growth factor A. Dexamethasone (10^−8^ M), β-glycerol phosphate (10 mM), and ascorbic acid (50 µg/mL) in DMEM constituted the conditioned medium for osteogenesis 24 h after lipofection. The cultured transgenic cells were fixed with methanol and rinsed with deionized water. The mineralized nodules were stained with 1% alizarin red S. The staining reaction was terminated by washing with water.

All the transgenic cells showed less ALP-positive staining intensities at days 7 and 14, which approached the control (no transgene) level by day 28 (Figure 2). Mineralized nodule formation was visible in all the groups at day 21 (data not shown) but was more prominent in all the transgenic cells, as shown in Figure 2. However, continuous loading of dexamethasone, β-glycerol phosphate, and ascorbic acid in the culture medium reduced the mineralization in all the transgenic cells by day 21 (Figure 3).

**Figure 4.**
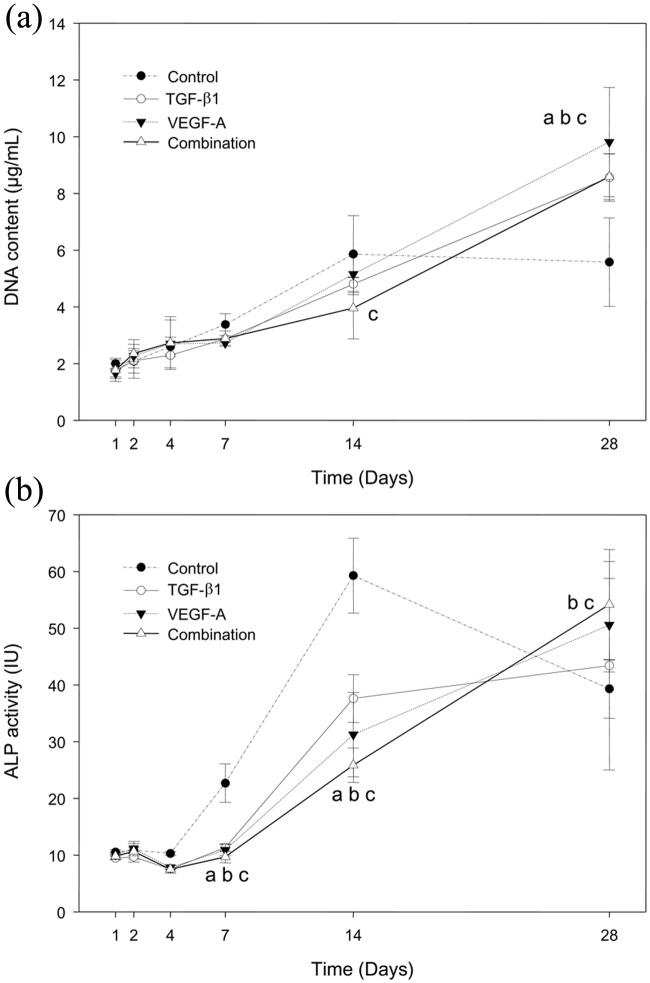
Cell proliferation and ALP activity. ALP: alkaline phosphatase; TGF-β1: transforming growth factor-beta 1; VEGF-A: vascular endothelial growth factor A. (a) The DNA amount was calculated by measuring the fluorescence of Hoechst dye, (b) the total ALP activity was determined by measuring the *p*-nitrophenol amount, and (c) the ALP activity was expressed as a ratio to the DNA amount. *p* < 0.05 indicates a significant difference (a: mTGF-β1 vs control; b: mVEGF-A vs control; c: mTGF-β1/mVEGF-A vs control).

Fluorometric assay of DNA showed excessive proliferation of the transgenic cells by day 28, whereas proliferation of the control cells reached a plateau level at day 14 (Figure 4(a)). In terms of the ALP activity, all the transgenic groups had higher levels of activity at day 28, whereas the activity in the control group was higher at day 7, peaked at day 14, and decreased thereafter to a level lower than that in all the transgenic groups (Figure 4(b)).

### Osteogenic gene expressions

**Figure 5.**
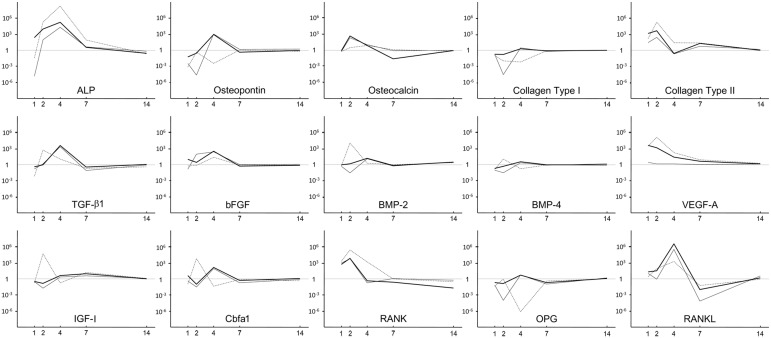
Profiling of the expressions of 15 osteogenic genes at days 1, 2, 4, 7, and 14 in vitro. ALP: alkaline phosphatase; TGF-β1: transforming growth factor-beta 1; VEGF-A: vascular endothelial growth factor A; bFGF: basic fibroblast growth factor; BMP-2: bone morphogenetic protein 2; BMP-4: bone morphogenetic protein 4; IGF-I: insulin-like growth factor I; RANK: receptor activator of nucleic factor kappa B; RANKL: receptor activator of nucleic factor kappa B ligand; Cbfa1: core binding factor alpha 1; OPG: Osteoprotegerin. cDNAs were reverse-transcribed and amplified by real-time PCR. The progress of the reaction was monitored by using fluorescence dye. The gene expression level in pooled samples (*n* = 3) from each experimental group was correlated with the cycle number at which the fluorescence reached the threshold. The expression level of each gene at a given time point was calculated by normalizing the signal to GAPDH gene expression and then expressed on a logarithmic scale as a fold change relative to the gene expression in the control culture.

As seen in Figure 5, the expressions of most of the osteogenic genes were stimulated at day 2 or 4 after immediate inhibition at day 1. All the transgenes increased the gene expression levels of ALP, rat TGF-β1 (rTGF-β1), basic fibroblast growth factor (bFGF), bone morphogenetic protein 2 (BMP-2), receptor activator of nucleic factor kappa B (RANK), receptor activator of nucleic factor kappa B ligand (RANKL), and runt-related gene 2/core binding factor alpha 1 (*Runx2*/Cbfa1) by 10 times or more than the expressions in the control group by day 2 or 4. Although osteocalcin (OC) gene expression was stimulated by less than tenfold in the mVEGF-A transgenic group at day 4, its expression prominently increased in the mTGF-β1 and mTGF-β1/mVEGF-A transgenic groups at day 2. Osteopontin (OP) gene expression was generally inhibited by the transgenes except for strong elevation in the mTGF-β1 and mTGF-β1/mVEGF-A transgenic groups at day 4. Unexpectedly, *BMP-4* gene expression was either upregulated or downregulated and affected in an opposite manner in the transgenic cells at days 2 and 4. The expression profile of insulin-like growth factor I (IGF-I) was similar to that of BMP-4 until day 4 but its expression level in all the transgenic groups was still slightly elevated at day 7. The gene expression level of rat VEGF-A (rVEGF-A) was not greatly affected except at day 1. Interestingly, the gene expression of collagen type II (COL-II) was extremely stimulated until day 7 in all the transgenic groups, whereas that of collagen type I (COL-I) had a completely opposite expression profile. Osteoprotegerin/osteoclastogenesis inhibitory factor (OPG/OCIF) gene expression almost countered RANK transcription over time. The expression levels of all the osteogenic genes converged to the control level by day 14.

### Composite implants

**Figure 6.**
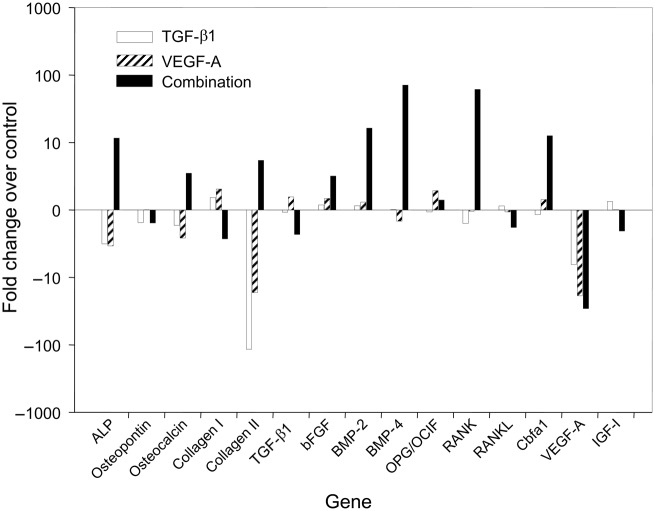
Real-time PCR analysis of the 15 osteogenic genes at day 14 in vivo. ALP: alkaline phosphatase; TGF-β1: transforming growth factor-beta 1; VEGF-A: vascular endothelial growth factor A; bFGF: basic fibroblast growth factor; BMP-2: bone morphogenetic protein 2; BMP-4: bone morphogenetic protein 4; IGF-I: insulin-like growth factor I; RANK: receptor activator of nucleic factor kappa B; RANKL: receptor activator of nucleic factor kappa B ligand; Cbfa1: core binding factor alpha 1; OPG/OCIF: Osteoprotegerin/osteoclastogenesis inhibitory factor; PCR: polymerase chain reaction; mRNA: messenger RNA. Pooled samples (*n* = 4) from each experimental group were assessed for mRNA amplification. The values are indicated as the mean relative to those for each gene expression in the control group on a logarithmic scale.

**Figure 7.**
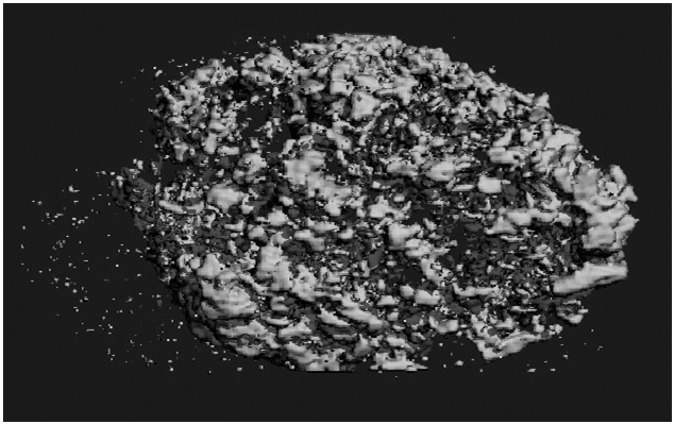
An µCT image of a collagen sponge. µCT: microcomputed tomography. The image is an example showing new apposition of mineral in the biomaterial. There was no significant difference in the mineral density and mineral content among the experimental groups.

**Figure 8.**
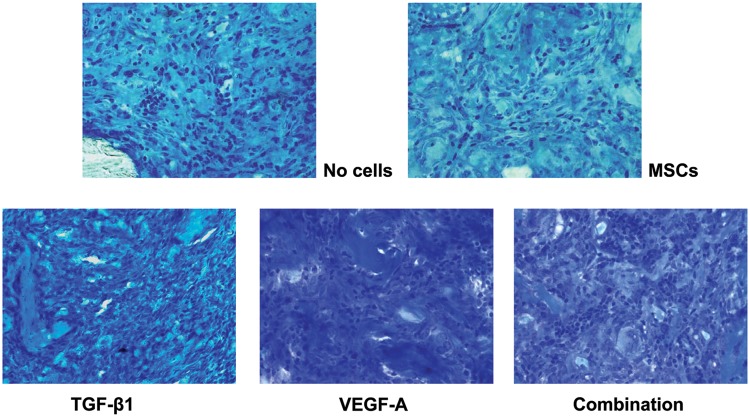
Histological view of the collagen scaffolds with the transgenic cells. TGF-β1: transforming growth factor-beta 1; VEGF-A: vascular endothelial growth factor A. Samples were embedded in paraffin and sectioned. The tissues were visualized by toluidine blue staining. Condensation of cells in the developing tissue in all the transgenic groups was more prominent upon histological examination.

As represented in Figure 5, the expression levels of many osteogenic genes in the implanted collagen scaffolds increased in the mTGF-β1, mVEGF-A, and mTGF-β1/mVEGF-A transgenic groups (Figure 6). Especially, the mTGF-β1/mVEGF-A transgenic group showed more than tenfold gene expression levels of ALP, BMP-2, BMP-4, RANK, and Runx2 compared with the control group. However, the expressions of ALP, OC, and COL-II were downregulated in the mTGF-β1 and mVEGF-A transgenic groups, with COL-II showing the greatest response. r*VEGF-A* gene expression was downregulated in all the groups by more than sixfold. The µCT analysis did not bring quantitative information of bone volume over tissue volume (BV/TV) and other histomorphometric parameters among the groups at day 14 (data not shown) because of contraction and unevenness of the samples, although new mineral apposition was observed in all the groups (Figure 7); however, paraffin-embedded histological sections demonstrated greater cell invasion within the residual fibrous tissue in the three transgenic groups (Figure 8).

## Discussion

This study demonstrated the effects of external application of *TGF-β1* and *VEGF-A* as transgenes in a rat bone marrow stromal cell culture and in vivo. The exogenous messenger RNA (mRNA) expressions could be distinguished from the endogenous r*TGF-β1* and r*VEGF-A* gene expressions by the use of specific primer sets. The method for gene transfer was lipofection with plasmid vectors encoding the m*TGF-β1* and m*VEGF-A* genes, and it was interesting to clarify how transcription of the encoded genes would be sustained and influence molecular or cellular events. Chemical-based transfection represented by lipofection has been outstripped by viral methods in terms of transfection efficacy and sustained release of the translated proteins.^30,31^ However, lipofection may be valuable as a nonviral method ensuring safety and ease of application, because chemical-based techniques such as lipofection and Ca-P precipitation generally provide mild expression of exogenous proteins carried as transgenes and their rapid degradation in the blood stream during cell segmentation with less probability of oncogenesis.

To observe transcription of the m*TGF-β1* and m*VEGF-A* genes, their expressions were analyzed quantitatively by real-time PCR. The expressions gradually disappeared within 7 days after transfection, coinciding with the fading of EGFP fluorescence (data not shown). Therefore, although the protein levels of mTGF-β and mVEGF-A were not examined because of very low specificity of their antibodies, which may also be susceptible to rTGF-β1/rVEGF-A, we consider that mTGF-β1 and mVEGF-A protein secretion decreased in the same manner as that of EGFP. These results suggest that the plasmid-based gene transfer into rat bone marrow stromal cells by lipofection could maintain the transcription and translation of the encoded genes for 7 days, followed thereafter by a gradual decrease.

On the other hand, the 15 osteogenic genes examined in the cell culture after the gene transfer had different expression profiles, gradually responding to the transgenes with a peak at about day 7. The gene-carrying concept used in this study was lipofection of plasmid vectors, which is known as a conventional method but does not function for continuous or long-term transcription as well as viral transfections do. Therefore, efficacy of the transgenes could only show positive or negative peaks for driving osteogenesis in the cell culture at an early time; however, it is hypothesized that this initiation to the cells derived from bone marrow might have affected osteoblastogenesis in an autocrine and an paracrine manners. Although plasmid-based gene transfer has poor efficiency and has been modified with biocompatible matrices in vivo,^14,15,32–34^ endogenous gene transcription could be maintained effectively for a couple of weeks in response to the exogenous genes in vitro. This result indicates that gene transfer can be considered and designed not only for the carried genes but also for a cascade of altered endogenous genes.

TGF-β enhances the proliferation and early differentiation of osteoblasts, which are the processes characterized by a high rate of collagen synthesis, but impairs their differentiation based on OC synthesis and mineralization of bone matrix.^35^ VEGF-A regulates human osteoblast differentiation in addition to its effects on osteoblast migration and proliferation.^36^ Another study has suggested that VEGF does not stimulate fetal bovine osteoblast proliferation but induces migration, parathyroid hormone (PTH)–dependent cyclic adenosine monophosphate (cAMP) accumulation, and ALP increase in this cell culture.^37^ Although the bone marrow stromal cells used in this study were derived from a different species (i.e. rats), they showed significantly enhanced proliferation influenced by either TGF-β1 or VEGF-A, carried as transgenes, as clarified by the increase in DNA synthesis in the fluorometric assay.

Both the transgenes temporally stimulated the expressions of 15 osteogenic genes including ALP. However, these transgenes repressed ALP activity significantly at days 7 and 14 and also reduced the intensity of ALP-positive cell staining at the same time points, followed by recovery of both ALP activity and ALP-positive cell staining close to the control levels at day 28 as the DNA synthesis increased. Therefore, the transgenic treatments may have regulated a posttranscriptional process of the ALP gene. However, mineralized nodule formation became more prominent in all the transgenic groups by day 28. Consequently, either the m*TGF-β1* or m*VEGF-A* gene might have extended the cell proliferation stage and delayed ALP expression; the increase in mineralized nodule formation might be attributed to a change in transcription and/or a posttranscriptional process of the transgenes, which are expected to promote and enhance osteogenic differentiation.

A recent study has demonstrated that recombinant TGF-β1 does not influence gene expression or protein production of OC and ALP but dramatically abolishes BMP-2-mediated OC gene expression and ALP activity, inhibiting the ability of BMP-2 to induce mineralization in murine cell lines.^38^ On the contrary, we found that although TGF-β1 delivery in rat bone marrow stromal cell culture by using a plasmid-repressed ALP gene expression at day 1 and ALP activity until day 14, mineralized nodule formation was finally enhanced with increased ALP gene expression, recovery of ALP activity at day 28, and elevated OC gene expression at days 2 and 4. The enhanced mineralized nodule formation could also be explained by the upregulated expressions of other genes at the early time points (days 2–7), such as OP, bFGF, rTGF-β1, BMP-2, BMP-4, Runx2, and IGF-I.

Interestingly, our study showed that *VEGF-A* gene transfer also stimulated mineralized nodule formation. As previously reported, VEGF expression is regulated by several growth factors, hormones, and cytokines including IGF-I and TGF-β1.^29,39^ However, the effects of VEGF on the induction of osteogenic gene expressions are still not clear. We noted that the m*VEGF-A* gene induced a similar change in most of the examined osteogenic genes as the m*TGF-β1* gene, including IGF-I and rTGF-β1 stimulation at an early time point (day 2). *VEGF* gene transfection dramatically stimulated rTGF-β1, BMP-2, BMP-4, and IGF-I mRNA expressions in addition to r*VEGF-A* gene expression, which was also enhanced by *TGF-β1* gene transfer in rat bone marrow stromal cells. Therefore, VEGF might contribute to the endogenous upregulation of gene expressions of TGF-β superfamilies, especially at early time points (days 2 and 4).

Signals downstream of VEGF and TGF-β interact and cross talk in a number of significant ways, acting either in a synergistic or antagonistic manner.^40^ Although all genes profiled in this study were not responded by the transgenes in a consistent manner, the combination treatment demonstrated acceleration of osteogenesis: for example, some genes such as ALP, OC, and COL-II were downregulated by each gene but upregulated by the combination and others were influenced synergistically or in different patterns.

In the ossifying cultures, dexamethasone (10^−8^ M), β-glycerol phosphate (10 mM), and ascorbic acid (50 µg/mL) were continuously added to promote osteoblastic differentiation of rat bone marrow stromal cells following the transgenic treatments. Similar to a previous report,^41^ our in vitro study showed that the set of osteogenic supplements in the medium at the given concentrations induced more mineralization than did the normal medium in the nontransgenic group; on the other hand, interestingly, all the transgenes dramatically inhibited mineralized nodule formation in the conditioned medium. Dexamethasone (10^−8^ M) with β-glycerol phosphate (10 mM) and ascorbic acid (50 µg/mL) increases ALP activity and collagen synthesis and induces abundant mineralization,^42,43^ accompanied with high expression of BMP-2.^41^ However, the negative effects of higher doses of glucocorticoids on bone include decreased bone formation, increased osteoclastic resorption, impaired calcium metabolism, and decreased production of sex steroids,^44–46^ whose direct effects include inhibition of cell cycle progression,^47,48^ induction of apoptosis,^49,50^ and impairment of osteoblast function through transcriptional and/or posttranscriptional inhibition of collagen,^51^ Runx2,^52^ and IGF-I and TGF-β.^44^ Furthermore, recombinant TGF-β1 added to confluent cultures of fetal rat calvarial cells with ascorbic acid and β-glycerol phosphate inhibits the formation of bone nodules.^53^ Taken together with our finding of increased r*TGF-β1* and r*VEGF-A* gene expressions by the transgenes, enhancing mineralized nodule formation, we suggest the following: (1) the osteogenic growth factors might have been stimulated by supplying the appropriate dose of dexamethasone in the culture medium, although we did not determine the ideal dose to upregulate these growth factors; (2) however, the mTGF-β1 and/or mVEGF-A loading into the medium containing dexamethasone, ascorbic acid, and β-glycerol phosphate was so high that not only transcription but also ALP activity and mineralized nodule formation were inhibited in a counter effect.

In vivo gene expressions in the bioreplaceable collagen scaffolds were also affected by both the m*TGF-β1* and m*VEGF-A* genes. Although the transgenes individually stimulated the expressions of several osteogenic genes, to less than tenfold, the fold change induced by the mTGF-β1/mVEGF-A combination was greater in many genes, suggesting that the dual-transgenic rat bone marrow stromal cells had upregulated expressions of genes involved in osteogenesis. Interestingly, the COL-I and COL-II gene expression levels showed the opposite response, which might indicate different roles of the transgenes in osteogenesis. Considering that the transgenes strongly stimulated RANK and RANKL gene expressions in the rat bone marrow stromal cells at the early time points until day 7 and greatly inhibited OPG/OCIF gene expression, *TGF-β1* or *VEGF-A* gene transfer is likely to be an important tool not only for bone formation but also for osteoclastogenesis triggering bone remodeling. Although no difference in bone formation was detected among the groups by µCT at day 14, the altered gene expressions can be expected to cause bone regeneration at a later time point. Unfortunately, the carrier of collagen sponge in this study could not be maintained stable underneath the skin and was decomposed after a couple of weeks.

In conclusion, the results of this study demonstrate that exogenous TGF-β1 and VEGF-A applied as transgenes have mitogenic effects on and enhance osteoblastic differentiation of bone marrow stromal cells and act synergistically. The data on cell behavior and gene expression profiles provide valuable information to optimize protocols for cell-based tissue engineering.

## References

[1] Caplan AI Mesenchymal stem cells. J Orthop Res 1991; 9: 641–650 1870029 10.1002/jor.1100090504

[2] Goshima J Goldberg VM Caplan AI The osteogenic potential of culture-expanded rat marrow mesenchymal cells assayed in vivo in calcium phosphate ceramic blocks. Clin Orthop Relat Res 1991; 262: 298–311 1984928

[3] Ohgushi H Goldberg VM Caplan AI Heterotopic osteogenesis in porous ceramics induced by marrow cells. J Orthop Res 1989; 7: 568–578 2544711 10.1002/jor.1100070415

[4] Ohgushi H Goldberg VM Caplan AI Repair of bone defects with marrow cells and porous ceramic. Experiments in rats. Acta Orthop Scand 1989; 60: 334–339 2665415 10.3109/17453678909149289

[5] Nakahara H Bruder SP Goldberg VM In vivo osteochondrogenic potential of cultured cells derived from the periosteum. Clin Orthop Relat Res 1990; 259: 223–232 2208860

[6] Lucas PA Syftestad GT Caplan AI A water-soluble fraction from adult bone stimulates the differentiation of cartilage in explants of embryonic muscle. Differentiation 1988; 37: 47–52 3384225 10.1111/j.1432-0436.1988.tb00795.x

[7] Nathanson MA Hay ED Analysis of cartilage differentiation from skeletal muscle grown on bone matrix. I. Ultrastructural aspects. Dev Biol 1980; 78: 301–331 7409307 10.1016/0012-1606(80)90338-3

[8] Bab I Passi-Even L Gazit D Osteogenesis in in vivo diffusion chamber cultures of human marrow cells. Bone Miner 1988; 4: 373–386 3191291

[9] Herbertson A Aubin JE Dexamethasone alters the subpopulation make-up of rat bone marrow stromal cell cultures. J Bone Miner Res 1995; 10: 285–294 7754809 10.1002/jbmr.5650100216

[10] McCulloch CA Strugurescu M Hughes F Osteogenic progenitor cells in rat bone marrow stromal populations exhibit self-renewal in culture. Blood 1991; 77: 1906–1911 2018833

[11] Langer R Vacanti JP Tissue engineering. Science 1993; 260: 920–926 8493529 10.1126/science.8493529

[12] Langer R Drug delivery and targeting. Nature 1998; 392: 5–10 9579855

[13] Fu S Liesveld J Mobilization of hematopoietic stem cells. Blood Rev 2000; 14: 205–218 11124108 10.1054/blre.2000.0138

[14] Bonadio J Tissue engineering via local gene delivery. J Mol Med 2000; 78: 303–311 11001527 10.1007/s001090000118

[15] Bonadio J Tissue engineering via local gene delivery: update and future prospects for enhancing the technology. Adv Drug Deliv Rev 2000; 44: 185–194 11072114 10.1016/s0169-409x(00)00094-6

[16] Lew D Parker SE Latimer T Cancer gene therapy using plasmid DNA: pharmacokinetic study of DNA following injection in mice. Hum Gene Ther 1995; 6: 553–564 7578393 10.1089/hum.1995.6.5-553

[17] Bonewald LF Mundy GR Role of transforming growth factor beta in bone remodeling: a review. Connect Tissue Res 1989; 23: 201–208 2698314 10.3109/03008208909002418

[18] Centrella M McCarthy TL Canalis E Transforming growth factor beta is a bifunctional regulator of replication and collagen synthesis in osteoblast-enriched cell cultures from fetal rat bone. J Biol Chem 1987; 262: 2869–2874 3469200

[19] Robey PG Young MF Flanders KC Osteoblasts synthesize and respond to transforming growth factor-type beta (TGF-beta) in vitro. J Cell Biol 1987; 105: 457–463 3475276 10.1083/jcb.105.1.457 PMC2114927

[20] Linkhart TA Mohan S Baylink DJ Growth factors for bone growth and repair: IGF, TGF beta and BMP. Bone 1996; 19: 1S–12S 8830994 10.1016/s8756-3282(96)00138-x

[21] Sandberg MM Aro HT Vuorio EI Gene expression during bone repair. Clin Orthop Relat Res 1993; 289: 292–312 8472429

[22] Rosen D Miller SC DeLeon E Systemic administration of recombinant transforming growth factor beta 2 (rTGF-beta 2) stimulates parameters of cancellous bone formation in juvenile and adult rats. Bone 1994; 15: 355–359 8068458 10.1016/8756-3282(94)90300-x

[23] Joyce ME Jingushi S Bolander ME Transforming growth factor-beta in the regulation of fracture repair. Orthop Clin North Am 1990; 21: 199–209 2296458

[24] Lind M Schumacker B Soballe K Transforming growth factor-beta enhances fracture healing in rabbit tibiae. Acta Orthop Scand 1993; 64: 553–556 8237323 10.3109/17453679308993691

[25] Leung DW Cachianes G Kuang WJ Vascular endothelial growth factor is a secreted angiogenic mitogen. Science 1989; 246: 1306–1309 2479986 10.1126/science.2479986

[26] Ferrara N Leung DW Cachianes G Purification and cloning of vascular endothelial growth factor secreted by pituitary folliculostellate cells. Methods Enzymol 1991; 198: 391–405 1857232 10.1016/0076-6879(91)98040-d

[27] Collin-Osdoby P Role of vascular endothelial cells in bone biology. J Cell Biochem 1994; 55: 304–309 7962161 10.1002/jcb.240550306

[28] Harada S Nagy JA Sullivan KA Induction of vascular endothelial growth factor expression by prostaglandin E2 and E1 in osteoblasts. J Clin Invest 1994; 93: 2490–2496 8200985 10.1172/JCI117258 PMC294462

[29] Saadeh PB Mehrara BJ Steinbrech DS Transforming growth factor-beta1 modulates the expression of vascular endothelial growth factor by osteoblasts. Am J Physiol 1999; 277: C628–C637 10516092 10.1152/ajpcell.1999.277.4.C628

[30] McMahon JM Conroy S Lyons M Gene transfer into rat mesenchymal stem cells: a comparative study of viral and nonviral vectors. Stem Cells Dev 2006; 15: 87–96 16522166 10.1089/scd.2006.15.87

[31] Mazur W Ali NM Grinstead WC Lipofectin-mediated versus adenovirus-mediated gene transfer in vitro and in vivo: comparison of canine and porcine model systems. Coron Artery Dis 1994; 5: 779–786 7858769

[32] Ochiya T Takahama Y Nagahara S New delivery system for plasmid DNA in vivo using atelocollagen as a carrier material: the minipellet. Nat Med 1999; 5: 707–710 10371512 10.1038/9560

[33] Shea LD Smiley E Bonadio J DNA delivery from polymer matrices for tissue engineering. Nat Biotechnol 1999; 17: 551–554 10385318 10.1038/9853

[34] Bonadio J Smiley E Patil P Localized, direct plasmid gene delivery in vivo: prolonged therapy results in reproducible tissue regeneration. Nat Med 1999; 5: 753–759 10395319 10.1038/10473

[35] Heberden C Denis I Pointillart A TGF-beta and calcitriol. Gen Pharmacol 1998; 30: 145–151 9502167 10.1016/s0306-3623(97)00271-1

[36] Mayr-Wohlfart U Waltenberger J Hausser H Vascular endothelial growth factor stimulates chemotactic migration of primary human osteoblasts. Bone 2002; 30: 472–477 11882460 10.1016/s8756-3282(01)00690-1

[37] Midy V Plouet J Vasculotropin/vascular endothelial growth factor induces differentiation in cultured osteoblasts. Biochem Biophys Res Commun 1994; 199: 380–386 8123039 10.1006/bbrc.1994.1240

[38] Spinella-Jaegle S Roman-Roman S Faucheu C Opposite effects of bone morphogenetic protein-2 and transforming growth factor-beta1 on osteoblast differentiation. Bone 2001; 29: 323–330 11595614 10.1016/s8756-3282(01)00580-4

[39] Goad DL Rubin J Wang H Enhanced expression of vascular endothelial growth factor in human SaOS-2 osteoblast-like cells and murine osteoblasts induced by insulin-like growth factor I. Endocrinology 1996; 137: 2262–2268 8641174 10.1210/endo.137.6.8641174

[40] Holderfield MT Hughes CC Crosstalk between vascular endothelial growth factor, notch, and transforming growth factor-beta in vascular morphogenesis. Circ Res 2008; 102: 637–652 18369162 10.1161/CIRCRESAHA.107.167171

[41] Bi LX Simmons DJ Mainous E Expression of BMP-2 by rat bone marrow stromal cells in culture. Calcif Tissue Int 1999; 64: 63–68 9868286 10.1007/s002239900580

[42] Green E Todd B Heath D Mechanism of glucocorticoid regulation of alkaline phosphatase gene expression in osteoblast-like cells. Eur J Biochem 1990; 188: 147–153 2318198 10.1111/j.1432-1033.1990.tb15382.x

[43] Ter Brugge PJ Jansen JA In vitro osteogenic differentiation of rat bone marrow cells subcultured with and without dexamethasone. Tissue Eng 2002; 8: 321–331 12031120 10.1089/107632702753725076

[44] Canalis E Delany AM Mechanisms of glucocorticoid action in bone. Ann N Y Acad Sci 2002; 966: 73–81 12114261 10.1111/j.1749-6632.2002.tb04204.x

[45] Luppen CA Leclerc N Noh T Brief bone morphogenetic protein 2 treatment of glucocorticoid-inhibited MC3T3-E1 osteoblasts rescues commitment-associated cell cycle and mineralization without alteration of Runx2. J Biol Chem 2003; 278: 44995–45003 12933820 10.1074/jbc.M306730200

[46] Luppen CA Smith E Spevak L Bone morphogenetic protein-2 restores mineralization in glucocorticoid-inhibited MC3T3-E1 osteoblast cultures. J Bone Miner Res 2003; 18: 1186–1197 12854828 10.1359/jbmr.2003.18.7.1186

[47] Chen TL Cone CM Feldman D Glucocorticoid modulation of cell proliferation in cultured osteoblast-like bone cells: differences between rat and mouse. Endocrinology 1983; 112: 1739–1745 6832067 10.1210/endo-112-5-1739

[48] Chevalley T Strong DD Mohan S Evidence for a role for insulin-like growth factor binding proteins in glucocorticoid inhibition of normal human osteoblast-like cell proliferation. Eur J Endocrinol 1996; 134: 591–601 8664980 10.1530/eje.0.1340591

[49] Smith E Redman RA Logg CR Glucocorticoids inhibit developmental stage-specific osteoblast cell cycle. Dissociation of cyclin A-cyclin-dependent kinase 2 from E2F4-p130 complexes. J Biol Chem 2000; 275: 19992–20001 10867026 10.1074/jbc.M001758200

[50] Weinstein RS Jilka RL Parfitt AM Inhibition of osteoblastogenesis and promotion of apoptosis of osteoblasts and osteocytes by glucocorticoids. Potential mechanisms of their deleterious effects on bone. J Clin Invest 1998; 102: 274–282 9664068 10.1172/JCI2799 PMC508885

[51] Delany AM Gabbitas BY Canalis E Cortisol downregulates osteoblast alpha 1 (I) procollagen mRNA by transcriptional and posttranscriptional mechanisms. J Cell Biochem 1995; 57: 488–494 7768983 10.1002/jcb.240570314

[52] Chang DJ Ji C Kim KK Reduction in transforming growth factor beta receptor I expression and transcription factor CBFa1 on bone cells by glucocorticoid. J Biol Chem 1998; 273: 4892–4896 9478931 10.1074/jbc.273.9.4892

[53] Harris SE Bonewald LF Harris MA Effects of transforming growth factor beta on bone nodule formation and expression of bone morphogenetic protein 2, osteocalcin, osteopontin, alkaline phosphatase, and type I collagen mRNA in long-term cultures of fetal rat calvarial osteoblasts. J Bone Miner Res 1994; 9: 855–863 8079661 10.1002/jbmr.5650090611

